# Appetite ratings and ghrelin concentrations in young adults after administration of a balanced meal. Does sex matter?

**DOI:** 10.1186/s13293-022-00434-2

**Published:** 2022-06-04

**Authors:** Alessandro Leone, Ramona De Amicis, Marta Pellizzari, Simona Bertoli, Simone Ravella, Alberto Battezzati

**Affiliations:** 1 International Center for the Assessment of Nutritional Status (ICANS), Department of Food, Environmental and Nutritional Sciences (DeFENS), University of Milan, Via Sandro Botticelli 21, 20133 Milan, Italy; 2 Lab of Nutrition and Obesity Research, Istituto Auxologico Italiano, IRCCS, Milan, Italy

**Keywords:** Sex differences, Mixed meal, Ghrelin, Satiety, Hunger, Energy expenditure

## Abstract

**Background:**

Sex-based differences in appetite ratings have been observed previously. Ghrelin is the only known orexigenic peptide hormone. Sex differences in postprandial ghrelin responses may underlie different perceptions of hunger and satiety, but results are conflicting. We conducted a parallel study to evaluate sex differences in postprandial appetite ratings and ghrelin concentration after administration of a physiological meal among students of University of Milan.

**Methods:**

Twenty-four healthy, normal weight volunteers (12 men and 12 women) aged 18–35 years were recruited. A balanced mixed meal meeting 40% of the estimated daily energy expenditure and providing 60% of calories from carbohydrates, 25% from lipids and 15% from protein was administrated. Sex differences in appetite ratings (satiety, hunger, fullness and desire to eat) and magnitude of ghrelin suppression during postprandial period (up to 180 min) were determined.

**Results:**

In the fasting state, men and women did not differ in appetite ratings and ghrelin concentrations. After feeding, women tended to reach peak of satiety earlier than men, who in turn reached the nadir of hunger later than women (median: 30 min, interquartile range (IQR): 1; 120 vs. 1 min, IQR 1; 1, *p* = 0.007). Ghrelin suppression was greater in women (median decremental AUC − 95, IQR − 122; − 66) than in men (median decremental AUC − 47, IQR − 87; − 31, *p* = 0.041).

**Conclusions:**

These findings suggest sex differences in the postprandial appetite regulation that might be important for nutritional strategy to prevent and treat obesity and eating disorders.

**Supplementary Information:**

The online version contains supplementary material available at 10.1186/s13293-022-00434-2.

## Highlights

- After consuming a balanced mixed meal, men have delayed hunger suppression compared with women, who instead experience early satiety.
- After consuming a balanced mixed meal, ghrelin is suppressed more in women than in men.
- Sex should be considered in the design of nutritional strategies for the prevention and management of obesity and eating disorders.

**Supplementary Information:**

The online version contains supplementary material available at 10.1186/s13293-022-00434-2.

## Introduction

Appetite regulation is a complex process involving neural and humoral peripheral signals that interact with the central nervous system, in which the hypothalamus plays a pivotal role [1]. The signaling systems underlying appetite control include peripheral signals of hunger and satiety from the gastrointestinal system, which primarily include gastric motility and peptide release from enteroendocrine cells [2, 3], and signals of adiposity and energy homeostasis that influence eating behavior and food intake [4, 5]. Dysfunction of the signaling network underlying hunger, satiety, and metabolic status has been thought to be involved in the etiology of obesity and eating disorders [6].

Among the gut hormones involved in the appetite regulation, ghrelin is the only known orexigenic peptide hormone. It is produced mainly by cells in the stomach and proximal small intestine [2, 3], and plays a central role in the short-term feeding regulation, given its implication in the hunger perception [7]. Its plasma concentrations increase markedly during fasting and before meals and decrease rapidly during the postprandial phase [8, 9]. Results from an animal study suggest sex differences in the impact of ghrelin on food intake. It has been shown that ghrelin increased food intake significantly more in male and untreated ovariectomized female rats than in female intact and estradiol-treated ovariectomized rats, suggesting that estradiol inhibits the orexigenic action of ghrelin in females and opening up the possibility that higher concentrations of ghrelin are required in females than in males to induce the sensation of hunger [10]. According to this hypothesis, some studies found women having higher ghrelin concentrations than men in fasting state [11–13]. It is unclear, instead, whether sex affects the ghrelin response to a physiological meal. Some studies have documented women being more sensitive to overfeeding [14] and perceiving greater satiety and lower hunger after the meal consumption [15–17]. Thus, it seems plausible that there is a sex difference in gastrointestinal hormonal signaling of hunger and satiety after meal consumption. Higher postprandial concentrations of cholecystokinin (CCK), a satiety-stimulating gastrointestinal hormone, have been observed in women compared to men [18]. Moreover, changes in plasma CCK concentration were associated with a greater increment in fullness and decrement in hunger in women than in men [19]. We hypothesized that, similarly, there might be greater suppression in ghrelin secretion in women than in men.

To test this hypothesis, we measured postprandial ghrelin concentration and appetite ratings in young men and women after administration of a mixed balanced meal. The meal was standardized on individual daily energy expenditure in order to take into account the different body composition of men and women that may affect ghrelin concentrations [20–22]. If confirmed, sex differences in ghrelin response to a meal may explain the different eating behavior observed during mealtime between men and women, and have important implications for nutritional strategies for the prevention and treatment of obesity and eating disorders.

### Materials and methods

#### Subjects

Twenty-four (12 women and 12 men) volunteers were selected among students of the University of Milan. Recruitment and testing were carried out at the International Center for the Assessment of Nutritional Status (ICANS), University of Milan (Italy), between March and June 2018. Participants had to be aged 18–35 years, normal weight and apparently healthy. History of overweight or obesity, having a diagnosis of anosmia and dysgeusia, endocrine disease (i.e., hyper- or hypothyroidism and diabetes mellitus, polycystic ovary syndrome), eating disorder or any disease causing significant impairment of nutritional status (i.e., Crohn’s disease, neoplasia end-stage renal failure, cirrhosis, congestive heart failure and chronic infection), having consumed drugs affecting endocrine function in the previous 2 months, use of estroprogestinics, recent (< 1 month) occurrence of acute illness or injury and elite athleticism were reasons for exclusion from the study. This study was conducted according to the guidelines laid down in the Declaration of Helsinki. The ethics committee of the University of Milan gave a positive opinion on the study (protocol n. 32/17). Written informed consent was obtained from all participants.

### Experimental protocol

In the weeks preceding the study, the volunteers were invited to present themselves fasting at ICANS, in order to undergo a detailed medical examination and anthropometric assessment. Subjects were asked about the type and time spent on physical activity. Women were also asked to report the onset of last menses in order to determine the cycle phase. The volunteers were then organized into eight groups of three people each. The meal test studies were performed in different days distributed over several weeks. Each group participated in one of these study days. Women were allocated to the study days according to the menstrual phase, in order to participate during one of the days corresponding to their follicular phase.

On the evening before the test, we asked the subjects to eat a standardized dinner consisting of rice or pasta with olive oil and/or Parmesan cheese and/or tomato sauce, meat or fish, vegetables with olive oil, bread and fresh fruit, and to finish the dinner by 2100 h. After that time, volunteers were asked to refrain from eating. Water was the only drink allowed.

On the study days, volunteers were asked to report to ICANS at 0830 h in a fasting state. Upon arrival, they were seated in the room prepared for testing, where three stations equipped with everything needed to eat the meal and perform venous samplings had been set up, one for each volunteer in the group. A physician then applied an intravenous catheter into an antecubital vein. Baseline venous blood samples were obtained. A registered dietitian instructed the volunteers on the definitions of hunger, satiety, fullness and desire to eat, and invited them to report their baseline appetite ratings using a 100 mm Visual Analogue Scale (VAS) anchored at either ends with opposite statements (“not all” and “very much”). At 0900 h the test meal was provided and the volunteers were asked to consume the entire meal within 15 min. Venous blood samples were obtained every hour after the meal consumption up to 180 min in order to measure serum ghrelin. Appetite ratings were assessed at the end of the meal and at 30, 60, 90, 120 and 180 min. In order not to influence appetite ratings, volunteers were asked not to talk to each other about how they felt after consuming the meal. Throughout the test, the physician and dietitian remained in the room with the volunteers, managing the timing of blood draws, assisting the volunteers, and ensuring that the meal was entirely consumed on time and that the recommendation not to exchange comments about one's appetite sensations was followed.

### Test meal

The test meal consisted of a sandwich of white bread, ham, extra-virgin olive oil and tomato. The meal size was different for each participant, as it had to satisfy 40% of individual daily energy expenditure, estimated multiplying resting energy expenditure, obtained by Harris and Benedict equation [23], for the corresponding physical activity level [24]. The meal had a fixed nutrient composition. Approximately 60% of calories derived from carbohydrates, 25% from lipids and 15% from protein. The meal also provided about 9 g/1000 kcal of dietary fiber. Subjects were asked to consume the entire meal within 15 min.

### Anthropometric measurements

International standard procedures were followed to measure anthropometric measurements [25]. Body weight, height, circumferences and skinfolds were measured on subjects wearing only light underwear. Body weight was measured by a Column scale (Seca 700 balance, Seca Corporation, Hanover, MD, USA) to 100 g. Body height was measured to the nearest 0.1 cm using a vertical stadiometer. Body mass index was then calculated. Waist circumference was measured with a non-stretch tape applied horizontally midway between the lower rib margin and the superior anterior iliac spine taken to the nearest 0.5 cm. Bicipital, tricipital, subscapular and suprailiac skinfold thicknesses were measured by Holtain Tanner/Whitehouse skinfold calliper (Holtain Ltd, Crymych, Wales). Each skinfold-thickness measurement was taken 3 times and a mean was calculated [25]. Body density and fat mass were then calculated by the Durnin and Womersley method [26] and by the Siri’s formula [27], respectively.

### Laboratory analysis

Circulating ghrelin concentrations were measured at baseline and every 60 min up to 3 h using an enzymatic immunoassay kit (BioVendor, Cat. No. RA194063400R, RRID: AB_2895669). This assay is based on a double-antibody sandwich technique. The wells of the plate are coated with a monoclonal antibody specific to the C-terminal part of ghrelin. The acetylcholinesterase (AChE)–Fab’ conjugate (Tracer) which recognizes the N-terminal part of unacylated ghrelin is also added to the wells. Intra- and inter-assay variations were 6.3% and 7.0%, respectively. The limit of determination of this assay is 0.2 pg/ml.

### Statistical analysis

Sample size calculation was based on a preliminary analysis of previously published data [28], where we found that, after administration of the same meal we used here, men and women had a ghrelin suppression at 60 min of 0.31 ± 0.18 ng/ml and 0.49 ± 0.16 ng/ml, respectively. With 80% power and a 5% significance level, it was estimated that a sample of 24 volunteers (12 women and 12 men) was sufficient to detect a high effect (Cohen’s *d* = 1.06) in postprandial ghrelin responses among the sexes.

Continuous variables are presented as median and interquartile range (IQR), as several variables did not follow a normal distribution. Two-sample comparisons between men and women were made by rank-sum test. To test sex differences in the postprandial variations of ghrelin concentrations and appetite ratings, mixed-effects linear regression models were used. Sex (0 = woman, 1 = man), time and sex*time interaction were included as fixed-effect predictors and the patient as random effect. A *P* value < 0.05 was considered statistically significant. Statistical analysis was performed using STATA version 12.0 (StataCorp).

## Results

### Volunteers

A total of 12 men and 12 women (median age: 24 years, IQR 22; 26 years; median BMI: 21.9 kg/m^2^, IQR 20.3; 23.1 kg/m^2^) were involved in the study, and their characteristics are reported in Table 1. Men and women did not differ for age and BMI. As expected, waist circumference was greater in men, whereas the opposite was found for the percentage of body fat. Men had a greater daily energy expenditure than women.

**Table 1:** Characteristics of the volunteers

	Women		Men		*P* value
	Median	P25; P75	Median	P25; P75	
Age (years)	24	22; 26	23	21; 25	0.322
BMI (kg/m^2^)	21.7	20.3; 22.9	22.1	20.3; 23.1	0.729
Waist circumference (cm)	72.0	70.2; 75.0	78.6	77.7; 82.3	0.002
Body fat (%)	26.7	21.9; 30.7	14.6	11.8; 17.5	< 0.001
Resting energy expenditure (kcal)	1392	1352; 1448	1807	1740; 1844	< 0.001
Total energy expenditure (kcal)	2171	2109; 2258	2801	2697; 2858	< 0.001

P25 = 25th percentile, P75 = 75th percentile

### Appetite ratings

**Fig. 1:**
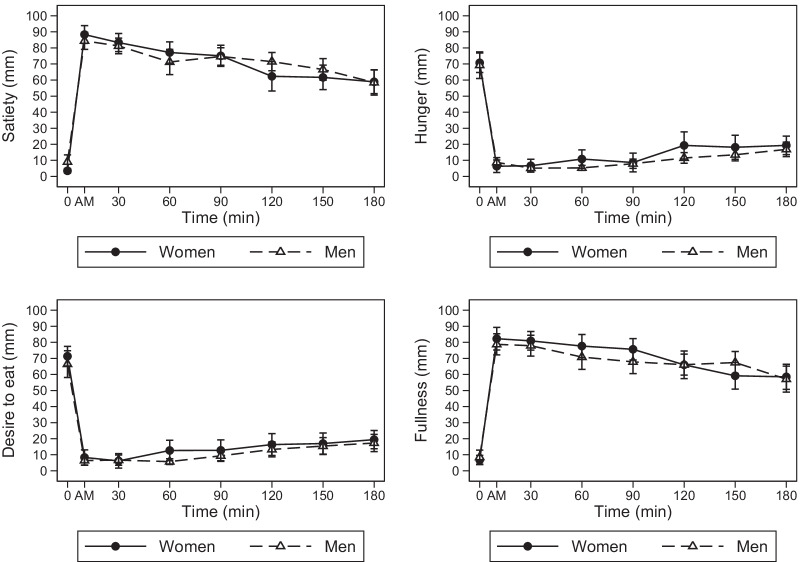
Appetite ratings (mean ± standard error) observed in men and women after ingestion of a balanced mixed meal

Appetite ratings over time according to sex are reported in Fig. 1.

Men and women had a similar perception of satiety, hunger, desire to eat and fullness at baseline (Table 2). In women, satiety and fullness peaked immediately after the consumption of the meal, increasing from baseline by 85 mm (95% CI 75; 94) and 76 mm (95% CI 65; 86), respectively. On the contrary, hunger reached the nadir immediately after the consumption of the meal, decreasing by 64 mm (95% CI 55; 74). Finally, the desire to eat reached the nadir at 30 min, decreasing by 65 mm (95% CI 56; 74). All appetite ratings were significantly different from baseline for the whole duration of the study. In men, appetite ratings changes over time from basal were not significantly different from those observed in women (sex*time interactions *p* > 0.05). Additional file 1: Table S1 shows the meal effect on appetite rating using men as reference.

**Table 2:** Meal effect on appetite rating according sex and time

	Satiety (mm)	Hunger (mm)	Desire to eat (mm)	Fullness (mm)
Sex^a^				
Men	5.58	− 1.50	− 4.92	1.75
	[− 11.32,22.48]	[− 15.34,12.34]	[− 19.22,9.39]	[− 16.59,20.09]
Time^b^				
1 min	84.92***	− 64.42***	− 63.00***	75.50***
	[75.42,94.41]	[− 73.81,− 55.02]	[− 72.31,− 53.69]	[65.11,85.89]
30 min	79.83***	− 64.08***	− 65.17***	74.17***
	[70.33,89.33]	[− 73.48,− 54.69]	[− 74.47,− 55.86]	[63.78,84.55]
60 min	73.67***	− 59.92***	− 58.67***	70.92***
	[64.17,83.17]	[− 69.31,− 50.52]	[− 67.97,− 49.36]	[60.53,81.30]
90 min	71.58***	− 62.08***	− 58.58***	68.92***
	[62.08,81.08]	[− 71.48,− 52.69]	[− 67.89,− 49.28]	[58.53,79.30]
120 min	58.83***	− 51.42***	− 55.00***	59.25***
	[49.33,68.33]	[− 60.81,− 42.02]	[− 64.31,− 45.69]	[48.86,69.64]
150 min	58.17***	− 52.58***	− 54.33***	52.42***
	[48.67,67.67]	[− 61.98,− 43.19]	[− 63.64,− 45.03]	[42.03,62.80]
180 min	55.42***	− 51.42***	− 51.92***	51.75***
	[45.92,64.92]	[− 60.81,− 42.02]	[− 61.22,− 42.61]	[41.36,62.14]
Sex*time interaction^c^				
Men*time (1 min)	− 9.58	3.92	3.00	− 5.25
	[− 23.01,3.84]	[− 9.37,17.20]	[− 10.16,16.16]	[− 19.94,9.44]
Men*time (30 min)	− 7.67	0.00	5.42	− 4.75
	[− 21.10,5.77]	[− 13.29,13.29]	[− 7.74,18.58]	[− 19.44,9.94]
Men*time (60 min)	− 11.50	− 4.00	− 2.00	− 8.58
	[− 24.93,1.93]	[− 17.29,9.29]	[− 15.16,11.16]	[− 23.27,6.11]
Men*time (90 min)	− 6.00	0.75	1.50	− 9.58
	[− 19.43,7.43]	[− 12.54,14.04]	[− 11.66,14.66]	[− 24.27,5.11]
Men*Time (120 min)	3.58	− 6.33	1.92	− 1.67
	[− 9.85,17.02]	[− 19.62,6.95]	[− 11.24,15.08]	[− 16.36,13.02]
Men*time (150 min)	− 0.67	− 3.17	3.33	6.50
	[− 14.10,12.77]	[− 16.45,10.12]	[− 9.83,16.49]	[− 8.19,21.19]
Men*time (180 min)	− 6.00	− 1.00	2.83	− 3.17
	[− 19.43,7.43]	[− 14.29,12.29]	[− 10.33,15.99]	[− 17.86,11.52]
Intercept^d^				
Constant	3.50	70.75***	71.33***	6.75
	[− 8.45,15.45]	[60.96,80.54]	[61.22,81.45]	[− 6.22,19.72]

Values are coefficients obtained from mixed-effects linear regression models

To obtain the mean values of the various appetite ratings over time, the coefficients shown in the table can be entered into the following sex-specific equations:

Women: constant + time (at a specific time point)

Men: constant + sex + time (at a specific time point) + interaction (at a specific time point)

^a^The effect of male sex on appetite ratings at baseline. It represents how much the basal ratings of appetite differed in men compared with women

^b^The effect of time on appetite ratings. It represents how much appetite ratings have changed over time from baseline in women

^c^The effect of male sex on appetite ratings over time. It represents how much the changes in appetite ratings from baseline in men differed from the changes observed in women over the same time frame

^d^The intercept represents the baseline appetite ratings in women

*p < 0.05, **p < 0.01, ***p < 0.001

However, we observed women reached the peak of satiety marginally earlier than men (*p* = 0.053, Cohen’s *d* = 0.83). Almost all women (92%) reported the highest satiety rating immediately after eating the meal, whereas 42% of men reported the peak of satiety at 30 min or later. On the contrary, the nadir of hunger was delayed in men (30 min, IQR 1; 120) than in women (1 min, IQR 1; 1, *p* = 0.007, Cohen’s d = 1.12) (Table 3).

**Table 3:** Postprandial appetite ratings parameters

	Women		Men		*P* value
	Median	P25; P75	Median	P25; P75	
Satiety					
Peak rating (mm)	98	85; 100	91	83; 100	0.514
Time to peak (min)	1	1; 1	1	1; 45	0.053
iAUC_0–180_	13,427	9062; 15,979	11,518	10,066; 13,244	0.488
tAUC_0–180_	14,507	10,163; 16,285	12,888	11,788; 15,086	1.000
Hunger					
Nadir rating (mm)	0	0; 2	1	0; 4	0.438
Time to nadir (min)	1	1; 1	30	1; 120	0.007
dAUC_0–180_	− 10,534	− 13,679; − 6470	− 11,634	− 13,630; − 8675	0.624
tAUC_0–180_	517	196; 3315	1808	347; 2232	0.817
Desire to eat					
Nadir rating (mm)	0	0; 5	0	0; 2	0.796
Time to nadir (min)	1	1; 30	30	1; 120	0.125
dAUC_0–180_	− 10,687	− 13,032; − 6888	− 10,474	− 1297; − 8278	1.000
tAUC_0–180_	507	190; 3866	1690	430; 2650	0.817
Fullness					
Peak rating (mm)	97	79; 100	85	83; 100	0.596
Time to peak (min)	1	1; 60	1	1; 45	0.923
iAUC_0–180_	11,783	8844; 15,331	11,337	9056; 13,000	0.564
tAUC_0–180_	14,705	9600; 16,174	12,919	11,147; 14,291	0.863

*P25* 25th percentile, *P75* 75th percentile, *iAUC* incremental area under the curve, *dAUC* decremental area under the curve, *tAUC* total area under the curve

### Ghrelin

**Fig. 2:**
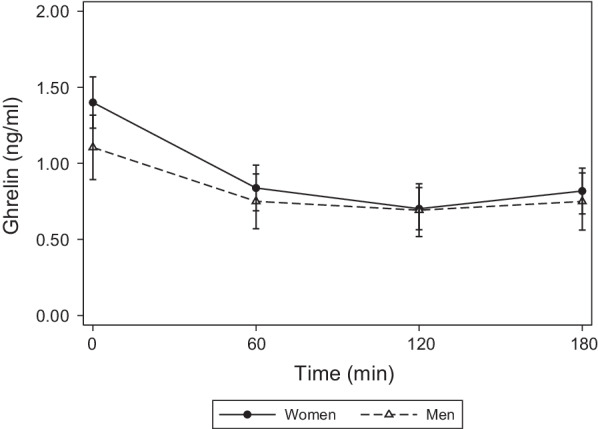
Ghrelin concentrations (mean ± standard error) observed in men and women after ingestion of a balanced mixed meal

Postprandial ghrelin concentrations according sex are reported in Fig. 2.

Fasting ghrelin concentrations were similar between men (1.1 ng/ml, IQR 0.28; 1.7) and women (1.4 ng/ml, IQR 1.2; 1.9; *p* = 0.406) (Table 4). In women, ghrelin significantly decreased from baseline by 0.56 ng/ml (95% CI − 0.68; − 0.44) at 60 min, by 0.70 ng/ml (95% CI − 0.82; − 0.58) at 120 min, and by 0.58 ng/ml (95% CI − 0.70; − 0.46) at 180 min (*η*^2^ time = 0.78), displaying a slight rebound increment. In men, ghrelin changes overtime from basal were significantly different from those observed in women (sex*time interaction *p* = 0.005, *η*^2^ = 0.18). The decrement was more accentuated in women than in men at each time point [0.21 ng/ml (95% CI − 0.38; − 0.04) greater suppression at 60 min, 0.29 ng/ml (95% CI − 0.45; − 0.12) at 120 min, and 0.23 ng/ml (95% CI − 0.39; − 0.06) at 180 min]. Additional file 1: Table S2 shows the meal effect on ghrelin concentrations using men as reference.

**Table 4:** Meal effect on ghrelin secretion according sex and time

	Ghrelin (ng/ml)
Sex^a^	
Men	− 0.30
	[− 0.75,0.16]
Time^b^	
60 min	− 0.56***
	[− 0.68,− 0.44]
120 min	− 0.70***
	[− 0.82,− 0.58]
180 min	− 0.58***
	[− 0.70,− 0.46]
Sex*time interaction^c^	
Men*Time (60 min)	0.21*
	[0.04,0.38]
Men*time (120 min)	0.29***
	[0.12,0.45]
Men*time (180 min)	0.23**
	[0.06,0.39]
Intercept^d^	
Constant	1.40***
	[1.08,1.72]

Values are coefficients obtained from mixed-effects linear regression models

To obtain the mean values of ghrelin over time, the coefficients shown in the table can be entered into the following sex-specific equations:

Women: constant + time (at a specific time point)

Men: constant + sex + time (at a specific time point) + interaction (at a specific time point)

^a^The effect of male sex on ghrelin at baseline. It represents how much the basal ghrelin concentration differed in men compared with women

^b^The effect of time on ghrelin concentration. It represents how much ghrelin concentration has changed over time from baseline in women

^c^The effect of male sex on ghrelin concentration over time. It represents how much the ghrelin changes from baseline in men differed from the changes observed in women over the same time frame

^d^The intercept represents the baseline ghrelin concentration in women

*p < 0.05, **p < 0.01, ***p < 0.001

Moreover, median decremental area over ghrelin response curve was greater in women than in men (− 95, IQR − 122; − 66 vs. − 47, IQR − 87; − 31, *p* = 0.041, Cohen’s *d* = 0.94) (Table 5).

**Table 5:** Parameters of postprandial ghrelin response

	Women		Men		*P* value
	Median	P25; P75	Median	P25; P75	
Nadir concentration (ng/ml)	0.7	0.3; 1.1	0.7	0.1; 1.2	1.000
Time to nadir (min)	120	60; 120	120	120; 120	0.111
dAUC_0–180_	− 95	− 122; − 66	− 47	− 88; − 31	0.041
tAUC_0–180_	144	105; 240	147	24; 242	0.762

*P25* 25th percentile, *P75* 75th percentile, *iAUC* incremental area under the curve, *dAUC* decremental area under the curve, *tAUC* total area under the curve

## Discussion

Our results show that men, after consuming a balanced mixed meal, have delayed hunger suppression compared with women, who instead experience early satiety. This result is supported, at least in part, by a greater reduction in circulating ghrelin concentrations in the three hours following meal consumption in women then in men, although there were no statistically significant sex differences in fasting ghrelin concentrations. Compared with previous studies that observed higher fasting ghrelin concentrations in women than in men [11–13], we observed no sex differences. This may be due to the fact that we conducted the study with women in the follicular phase of the cycle, when estrogen concentrations are low, and lesser could be the modulatory activity they have on ghrelin concentration [29, 30]. Our results also show that men and women experienced different subjective perceptions of hunger and satiety although the mean ghrelin values at the various times were similar. Currently, it is not possible to associate absolute levels of ghrelin concentration with subjective measures of appetite. In addition, ghrelin levels are confounded by many factors. The result is a large inter-subject variability in ghrelin levels that may not be associated with different perceptions of hunger and satiety. On the contrary, rapid variations in ghrelin within the individual provide a strong physiological signal related to subjective measures of satiety.

Our results in postprandial appetite ratings are in agreement with previous findings showing women to experience a greater satiety than men after an ad libitum consumption of a liquid or solid balanced mixed meal [15–17]. Several mechanisms have been proposed to explain this result. First, female sex hormones, particularly estrogen, are known to be implicated in the regulation of food intake, as they modulate responsiveness to anorectic hormones such as leptin, cholecystokinin, GLP-1, and peptide YY, both at the level of vagal afferent neurons and at the level of nuclei in the central nervous system [31], and therefore may have contributed to more rapid suppression of appetite in women. Second, it is possible that the meal resulted in a greater increment in CCK in women than in men [18]. The increase in postprandial CCK also appears to be associated with a greater increase in fullness and greater decrease in hunger in women than in men [19]. Note that these results were obtained by administering meals having 20–30% of calories from fat, similarly to the meal we provided. Third, several studies observed sex-specific brain areas activation in response to hunger and satiation [17, 32], as well as in response to food stimuli [17, 33], suggesting sex-specific differences in the cognitive and emotional processing of hunger and satiation. Additionally, our data show sex-related difference in postmeal ghrelin decrement which may furtherly explain the differences between men and women in the appetite regulation. This difference does not appear to be influenced by a different postprandial glucose metabolism between sexes. Insulin is known to influence circulating ghrelin levels. However, we recently found that men and women had the same glycemic, insulinemic, and C-peptidemic response by administering the same meal protocol presented in this study [34].

To the best of our knowledge, this is the first study reporting sex differences in ghrelin concentrations after consuming a balanced mixed meal. Previous studies regarding this topic were limited and the results conflicting. Carroll et al. [35] did not detect sex difference in ghrelin concentrations in the first 60 min after administration of an ad libitum liquid mixed meal with fixed nutrients amounts. Differently, Greenman et al. [36] observed higher ghrelin concentrations in women compared with men after administration of an oral load of glucose and lipids, but not protein. Our results differ from those reported in these studies and the first reason for this discrepancy may be in the meal administration protocol. It has been reported that men are more likely to overeat when asked to consume an ad libitum meal, whereas women are more likely to maintain an isocaloric intake [17]. The different amounts of food consumed may have influenced the pattern of postprandial hormones, preventing detection of sex differences. On the other hand, the standardization on individual energy needs of a whole mixed meal to be consumed, allows to study the postprandial physiological response of hormones influencing hunger and satiety. In addition, the effect observed by ingesting solutions containing single nutrients might not reflect the effect of nutrients within a complex matrix, as is the case of a meal. It has been observed that the ingestion of isocaloric beverages with one prevailing nutrient led to different results from those observed after single-nutrient oral load [37]. In the present study, we administer a meal complying the nutritional recommendations for a healthy, balanced meal [24] and therefore the results can be considered representative of reality. However, it cannot be excluded that a meal with a very different nutritional composition, or a vegetarian or vegan meal, which would contribute to higher fiber intake, may lead to different results. Finally, standardizing on individual energy expenditure controls for sex-related differences in body composition that could affect ghrelin concentrations. The second reason that might explain the discrepancy with previous studies is the homogeneity, in terms of age and nutritional status, of the subjects involved in the present study. Both appetite ratings and ghrelin concentrations have been found to change between age and BMI classes [15, 21, 38, 39]. Therefore, recruitment of subjects homogeneous in age and nutritional status allowed us to limit variability due to these factors.

Despite the peculiarities and strengths just described, we recognize that the present study is not without its limitations. Although the sample size calculation suggested that recruitment of 24 subjects was sufficient to detect a significant difference, this is still a small sample size. Although homogeneous recruitment by age and nutritional status limited the variability among subjects, this limits the generalizability of the results. It is not possible to transfer the results presented here to older or overweight or obese subjects without prior confirmation. A further limitation of the generalizability of these results is the recruitment of Caucasian subjects only. Indeed, ethnicity-related differences in postprandial ghrelin suppression have been reported [40], and thus these results need confirmation in subjects of non-Caucasian ethnicity. A further limitation of the study is that resting energy expenditure was estimated using the Harris & Benedict formula. However, we recently showed good agreement and low percent bias between predicted and measured resting energy expenditure in normal weight individuals [41]. A final limitation is that we did not measure leptin and other gastrointestinal hormones involved in the regulation of appetite, as well as we did not measure sex hormones. However, to reduce the variability associated with different concentrations of female sex hormones during different phases of the cycle, the study was conducted when the women were in the follicular phase of the menstrual cycle. Nevertheless, it should be reminded that estradiol levels vary significantly between each individual woman even within the same menstrual phase.

### Perspectives and significance

The sexual dimorphism observed in appetite perception and ghrelin suppression underlines the importance of potentially studying men and women as separate groups and of considering sex in the design of nutritional strategies for the prevention and management of obesity and eating disorders. Future studies on this issue that consider other population groups (elderly, obese, etc.) are strongly required.

## Conclusion

In conclusion, our results support the hypothesis of different appetite regulation between men and women, in part due to different postprandial ghrelin regulation. When a balanced mixed meal is ingested, women present a greater satiety immediately after the meal consumption, whereas men exhibit delayed hunger suppression, supported by a smaller decrease in postprandial ghrelin.

## Supplementary Information

**Additional file 1: Table S1.** Meal effect on appetite rating according sex and time (using men as reference). **Table S2.** Meal effect on ghrelin according sex and time (using men as reference) (DOCX 15 KB)

## Data Availability

The datasets used and/or analysed during the current study are available from the corresponding author on reasonable request.
