# Supplementary material for: Appetite ratings and ghrelin concentrations in young adults after administration of a balanced meal. Does sex matter?
Source: Biol Sex Differ. 2022 Jun 4;13:25. doi: 10.1186/s13293-022-00434-2 (PMC9167557; doi:10.1186/s13293-022-00434-2)
Supplement: Supplementary file 1 — Additional file 1: Table S1. Meal effect on appetite rating according sex and time (using men as reference). Table S2. Meal effect on ghrelin according sex and time (using men as reference) (DOCX 15 KB) [file 13293_2022_434_MOESM1_ESM.docx]

Table S1: Meal effect on appetite rating according sex and time (using men as reference)

|  | Satiety | Hunger | Desire | Fullness |
| --- | --- | --- | --- | --- |
| **Sex**^a^ |  |  |  |  |
| Women | -5.58 | 1.50 | 4.92 | -1.75 |
|  | [-22.49,11.32] | [-12.34,15.34] | [-9.39,19.22] | [-20.09,16.59] |
| **Time**^b^ |  |  |  |  |
| 1 min | 75.33*** | -60.50*** | -60.00*** | 70.25*** |
|  | [65.84,84.83] | [-69.89,-51.11] | [-69.31,-50.69] | [59.86,80.64] |
| 30 min | 72.17*** | -64.08*** | -59.75*** | 69.42*** |
|  | [62.67,81.66] | [-73.48,-54.69] | [-69.06,-50.44] | [59.03,79.80] |
| 60 min | 62.17*** | -63.92*** | -60.67*** | 62.33*** |
|  | [52.67,71.66] | [-73.31,-54.52] | [-69.97,-51.36] | [51.95,72.72] |
| 90 min | 65.58*** | -61.33*** | -57.08*** | 59.33*** |
|  | [56.09,75.08] | [-70.73,-51.94] | [-66.39,-47.78] | [48.95,69.72] |
| 120 min | 62.42*** | -57.75*** | -53.08*** | 57.58*** |
|  | [52.92,71.91] | [-67.14,-48.36] | [-62.39,-43.78] | [47.20,67.97] |
| 150 min | 57.50*** | -55.75*** | -51.00*** | 58.92*** |
|  | [48.00,67.00] | [-65.14,-46.36] | [-60.31,-41.69] | [48.53,69.30] |
| 180 min | 49.42*** | -52.42*** | -49.08*** | 48.58*** |
|  | [39.92,58.91] | [-61.81,-43.02] | [-58.39,-39.78] | [38.20,58.97] |
| **Sex*Time interaction**^c^ |  |  |  |  |
| Women*Time (1 min) | 9.58 | -3.92 | -3.00 | 5.25 |
|  | [-3.84,23.01] | [-17.20,9.37] | [-16.16,10.16] | [-9.44,19.94] |
| Women*Time (30 min) | 7.67 | -0.00 | -5.42 | 4.75 |
|  | [-5.76,21.09] | [-13.29,13.29] | [-18.58,7.74] | [-9.94,19.44] |
| Women*Time (60 min) | 11.50 | 4.00 | 2.00 | 8.58 |
|  | [-1.93,24.93] | [-9.29,17.29] | [-11.16,15.16] | [-6.11,23.27] |
| Women*Time (90 min) | 6.00 | -0.75 | -1.50 | 9.58 |
|  | [-7.43,19.43] | [-14.04,12.54] | [-14.66,11.66] | [-5.11,24.27] |
| Women*Time (120 min) | -3.58 | 6.33 | -1.92 | 1.67 |
|  | [-17.01,9.84] | [-6.95,19.62] | [-15.08,11.24] | [-13.02,16.36] |
| Women*Time (150 min) | 0.67 | 3.17 | -3.33 | -6.50 |
|  | [-12.76,14.09] | [-10.12,16.45] | [-16.49,9.83] | [-21.19,8.19] |
| Women*Time (180 min) | 6.00 | 1.00 | -2.83 | 3.17 |
|  | [-7.43,19.43] | [-12.29,14.29] | [-15.99,10.33] | [-11.52,17.86] |
| **Intercept**^d^ |  |  |  |  |
| Constant | 9.08 | 69.25*** | 66.42*** | 8.50 |
|  | [-2.87,21.03] | [59.46,79.04] | [56.30,76.53] | [-4.47,21.47] |

Values are coefficients obtained from mixed-effects linear regression models. Coefficients represents:

^a^ The effect of female sex on appetite ratings at baseline. It represents how much the basal ratings of appetite differed in women compared with men.

^b^ The effect of time on appetite ratings. It represents how much appetite ratings have changed over time from baseline in men.

^c^ The effect of female sex on appetite ratings over time. It represents how much the changes in appetite ratings from baseline in women differed from the changes observed in men over the same time frame.

^d^ The intercept represents the baseline appetite ratings in men.

To obtain the mean values of the various appetite ratings over time, the coefficients shown in the table can be entered into the following sex-specific equations:

Men: constant + time (at a specific time point)

Women: constant + sex + time (at a specific time point) + interaction (at a specific time point)

Abbreviations: *p<0.05 **p<0.01 ***p<0.001

Table S2: Meal effect on ghrelin according sex and time (using men as reference)

|  | Ghrelin |
| --- | --- |
| **Sex**^a^ |  |
| Women | 0.30 |
|  | [-0.16,0.75] |
| **Time**^b^ |  |
| 60 min | -0.35*** |
|  | [-0.47,-0.24] |
| 120 min | -0.41*** |
|  | [-0.53,-0.29] |
| 180 min | -0.36*** |
|  | [-0.47,-0.24] |
| **Sex*Time interaction**^c^ |  |
| Women*Time (60 min) | -0.21* |
|  | [-0.38,-0.04] |
| Women*Time (120 min) | -0.29*** |
|  | [-0.45,-0.12] |
| Women*Time (180 min) | -0.23** |
|  | [-0.39,-0.06] |
| **Intercept**^d^ |  |
| Constant | 1.10*** |
|  | [0.79,1.42] |

Values are coefficients obtained from mixed-effects linear regression models. Coefficients represents:

^a^ The effect of female sex on ghrelin at baseline. It represents how much the basal ghrelin concentration differed in women compared with men.

^b^ The effect of time on ghrelin concentration. It represents how much ghrelin concentration has changed over time from baseline in men.

^c^ The effect of female sex on ghrelin concentration over time. It represents how much the ghrelin changes from baseline in women differed from the changes observed in men over the same time frame.

^d^ the intercept represents the baseline ghrelin concentration in men.

To obtain the mean values of ghrelin over time, the coefficients shown in the table can be entered into the following sex-specific equations:

Men: constant + time (at a specific time point)

Women: constant + sex + time (at a specific time point) + interaction (at a specific time point)

Abbreviations: *p<0.05 **p<0.01 ***p<0.001
